# A pragmatic randomised controlled non-inferiority trial of open-door policy versus treatment as usual in urban psychiatric inpatient wards

**DOI:** 10.1192/j.eurpsy.2024.175

**Published:** 2024-08-27

**Authors:** A.-M. R. Indregard, M. S. Tesli, J. Gather, M. C. Småstuen, H. M. Nussle, P. O. Vandvik, N. Kunøe

**Affiliations:** ^1^Psychiatry, The Lovisenberg Diaconal Hospital; ^2^Health Services Research, Oslo Metropolitan University; ^3^Mental Health and Suicide, Norwegian Institute of Public Health; ^4^Regional Center, SIFER National Research Centre on Security, Prisons and Forensic Psychiatry, Oslo, Norway; ^5^Psychiatry, Psychotherapy and Preventive Medicine, LWL University Hospital, Ruhr University Bochum, Bochum, Germany; ^6^Nursing and Health Promotion, Oslo Metropolitan University; ^7^ University of Oslo; ^8^Department of Medicine; ^9^Department of Psychiatry, The Lovisenberg Diaconal Hospital, Oslo, Norway

## Abstract

**Introduction:**

Open-door policy (ODP) is an approach to reduce coercion in psychiatric wards recommended by the World Health Organization and the Council of Europe. Observational studies from Switzerland and Germany have shown promising results in reducing coercion, but no RCTs have been conducted. Skeptics have been concerned the observational evidence could mask that ODP could increase risks and harms and / or increase the use of coercive measures staff use to assist patients with psychoses, while proponents have argued that de-escalation and alliance-building will result in no such increase.

**Objectives:**

To evaluate open-door policy in an openly randomised, ethical-board approved trial of all patients referred to ward care at the Lovisenberg Diaconal Hospital in Oslo, Norway.

**Methods:**

A 12-month pragmatic, randomised-controlled non-inferiority trial comparing two ODP and three TAU acute psychiatric wards. The trial was pre-registered (ISRCTN16876467) and conformed to CONSORT. Ethical committee exemption enabled waiver of consent rules for the study, meaning all regular patients were included. Patients were randomly assigned (2:3 ratio) by a clinical admissions team using an open list. The non-inferiority margin was 15 % on the primary outcome: the proportion of patient stays with one or more coercive measures, including involuntary medication, isolation/seclusion, and physical and mechanical restraints. Primary and safety analyses were based on intention-to-treat. Safety analyses included suicides and violent events against staff. Secondary outcomes were individual coercive measures, intensive care, resource use, and patient feedback.

**Results:**

N=556 patients were included and randomised and were similar on all pre-admission demographics: Around 75% of patients were diagnosed with a psychotic disorder and were involuntarily admitted. Primary outcome: Use of coercive measures was within the non-inferiority margin (see table 1). Safety outcomes: No suicides occurred during ward care in any group. Violence against staff did not differ between study wards. Secondary outcomes: Use of intensive care (‘skjerming’) and number of days admitted was significantly less on open-door policy wards. Patients on open-door policy wards rated their experience of coercion and ward atmosphere better than patients on control wards.

**Table 1.** Absolute and relative risk of being subjected to coercion on open-door policy or usual-treatment wards.

	Number (%)				
Main outcome	Absolute Risk ODP wards (n=245)	Absolute Risk TAU wards (n=311)	Relative Risk (95% CI)	Risk Difference (95% CI)	Primary hypothesis confirmed
One or more coercive measures during the admission	65 (26.5%)	104 (33.4%)	1.3 (0.97 to 1.6)	6.9% (-0.7 to 14.5)	Yes

**Conclusions:**

This first RCT found open-door policy does not increase use of coercion or resource use. It does not harm staff or patients and is experienced as better by patients.

**Disclosure of Interest:**

None Declared

